# Supplementary material for: Usefulness of the Trabecular Bone Score in Assessing the Risk of Vertebral Fractures in Patients with Cirrhosis
Source: J Clin Med. 2022 Mar 12;11(6):1562. doi: 10.3390/jcm11061562 (PMC8954474; doi:10.3390/jcm11061562)
Supplement: Supplementary file 1 [file jcm-11-01562-s001.zip › jcm-1572738-supplementary.pdf]

Supplementary Table S1. Comparison of AUC between TBS and BMD

	TBS	BMD	*p*–value†
AUC (95% CI)	0.76 (0.69–0.83)	0.69 (0.61–0.77)	0.040

†AUC were compared using the Hanley-McNeil test.

AUC, area under the receiver operating characteristic curve; BMD, bone mineral density; CI, confidence interval; TBS, trabecular bone score

Supplementary Table S2. Association between bone microarchitecture structure grades and vertebral fractures

	Total	Men	Women
Predictors	OR† (95% CI)	OR† (95% CI)	OR† (95% CI)
Partially degraded structure‡	2.30 (1.14–4.64)	2.31 (0.66–8.09)	1.55 (0.63–3.78)
Degraded structure‡	15.17 (6.22–36.98)	24.55 (4.25–141.71)	8.44 (2.87–24.83)

†Estimated per one standard deviation decrease

‡Normal structure used as a reference

CI, confidence interval; OR, odds ratio

Supplementary Table S3. Association between trabecular bone score and vertebral fractures in patients not receiving osteoporosis medications.

	Total		Men		Women	
Predictors	OR† (95% CI)	AUC (95% CI)	OR† (95% CI)	AUC (95% CI)	OR† (95% CI)	AUC (95% CI)
TBS unadjusted	2.07 (1.58–2.71)	0.73 (0.64–0.82)	2.97 (1.41–6.28)	0.70 (0.57–0.82)	2.42 (1.51–3.89)	0.77 (0.63–0.91)
TBS adjusted for age	2.03 (1.52–2.70)	0.81 (0.73–0.88)	3.22 (1.40–7.40)	0.78 (0.67–0.89)	2.17 (1.31–3.60)	0.83 (0.72–0.93)
TBS adjusted for age and BMD	2.32 (1.62–3.32)	0.81 (0.74–0.89)	5.85 (2.01–17.06)	0.81 (0.70–0.92)	2.13 (1.24–3.67)	0.83 (0.73–0.93)
TBS adjusted for age, BMD and sex	2.77 (1.68–4.54)	0.81 (0.74–0.89)	N/A	N/A	N/A	N/A

†Estimated per one standard deviation decrease

AUC, area under the receiver operating characteristic curve; BMD, bone mineral density; CI, confidence interval; N/A, not available; OR, odds ratio; TBS, trabecular bone score

Supplementary Table S4. Association between trabecular bone score and vertebral fractures in subgroups adjusted for age, sex, and BMD

	Hepatocellular carcinoma		Other etiology		Liver decompensation	
Predictors	OR† (95% CI)	AUC (95% CI)	OR† (95% CI)	AUC (95% CI)	OR† (95% CI)	AUC (95% CI)
TBS unadjusted	2.38 (1.66–3.42)	0.80 (0.72–0.88)	2.21 (1.56–3.13)	0.76 (0.66–0.86)	3.11 (1.71–5.62)	0.69 (0.63–0.92)
TBS adjusted for age	2.28 (1.54–3.39)	0.84 (0.77–0.92)	2.24 (1.55–3.25)	0.84 (0.76–0.92)	2.78 (1.48–5.21)	0.90 (0.82–0.97)
TBS adjusted for age and BMD	2.26 (1.37–3.72)	0.84 (0.77–0.92)	2.32 (1.51–3.57)	0.84 (0.76–0.91)	2.08 (1.02–4.24)	0.90 (0.83–0.97)
TBS adjusted for age, BMD and sex	2.10 (1.16–3.79)	0.85 (0.77–0.92)	2.53 (1.46–4.39)	0.84 (0.76–0.92)	1.92 (0.81–4.61)	0.90 (0.82–0.97)

†Estimated per one standard deviation decrease

AUC, area under the receiver operating characteristic curve; BMD, bone mineral density; CI, confidence interval; OR, odds ratio; TBS, trabecular bone score

Supplementary Table S5. Factors associated with trabecular bone score among patients not administered osteoporosis medications†

Predictors	Partial regression coefficient (B)	Standard error	T-value	*p*-value	Standardized partial regression coefficient (β)	VIF
Total						
Age	−0.002	<0.001	−5.06	<0.001	−0.259	1.07
BMI	−0.006	0.001	−5.92	<0.001	−0.303	1.07
BMD	0.258	0.024	10.69	<0.001	0.551	1.09
Men						
Age	−0.002	<0.001	−3.44	<0.001	−0.238	1.12
BMI	−0.008	0.001	−6.05	<0.001	−0.405	1.05
BMD	0.228	0.028	8.07	<0.001	0.550	1.09
Women						
Age	−0.002	<0.001	−2.88	0.005	−0.266	1.24
BMI	−0.004	0.002	−2.34	0.022	−0.207	1.14
BMD	0.288	0.054	5.35	<0.001	0.507	1.31

†After adjustment for age, BMI, MELD score, calcium, 25(OH)D, and BMD, all of which were significant ($p < 0.05$) in univariate analysis

BMI, body mass index; BMD, bone mineral density; VIF, variance inflation factor

Supplementary Table S6. Factors associated with trabecular bone score among patients with and without HCC†

Predictors	Partial regression coefficient (B)	Standard error	T-value	*p*-value	Standardized partial regression coefficient (β)	VIF
Patients with HCC						
Age	−0.002	<0.001	−3.44	<0.001	−0.222	1.03
BMI	−0.005	0.001	−3.69	<0.001	−0.240	1.05
BMD	0.276	0.031	8.98	<0.001	0.587	1.06
Patients without HCC						
Age	−0.002	<0.001	−3.38	0.001	−0.240	1.11
BMI	−0.006	0.002	−3.76	<0.001	−0.266	1.10
BMD	0.251	0.031	7.97	<0.001	0.570	1.13

†After adjustment for age, BMI, MELD score, calcium, 25(OH)D, and BMD, all of which were significant ($p < 0.05$) in univariate analysis.

BMI, body mass index; BMD, bone mineral density; HCC, hepatocellular carcinoma; VIF, variance inflation factor

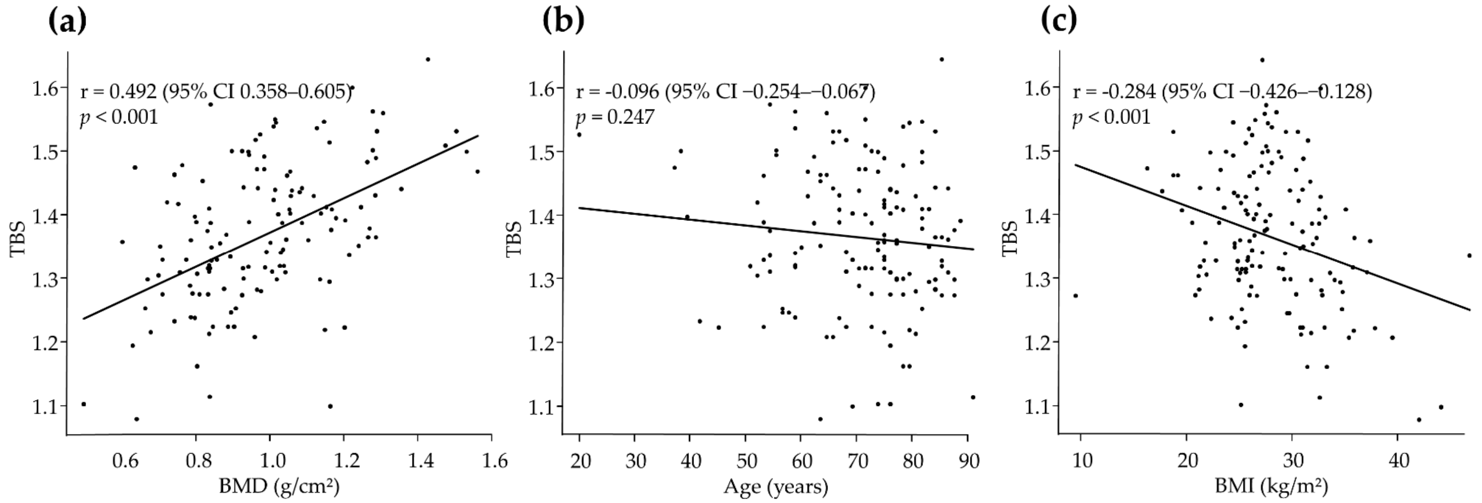

Supplementary Figure S1 Correlation coefficient in men between TBS and (a) BMD, (b) age, and (c) BMI. The correlation coefficient between TBS and BMD is positive (r = 0.492, $p < 0.001$), whereas the correlation is negative for BMI (r = −0.284, $p < 0.001$). No significant correlation is found between TBS and age (r = −0.096, $p = 0.247$). The correlation between TBS and the variables of interest is analyzed using the Pearson correlation coefficient.

BMD, bone mineral density; BMI, body mass index; TBS, trabecular bone score

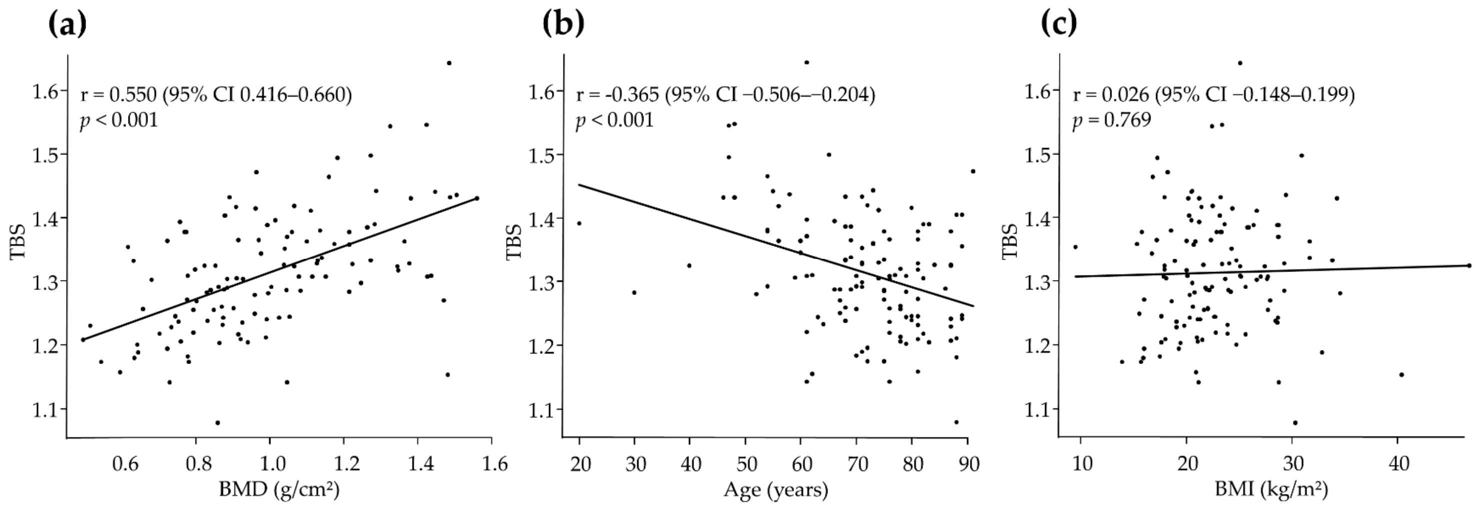

Supplementary Figure S2 Correlation coefficient in women between TBS and (a) BMD, (b) age, and (c) BMI. The correlation coefficient between TBS and BMD is positive (r = 0.550, p < 0.001), whereas the correlation is negative for age (r = −0.365, p < 0.001). No significant correlation is found between TBS and BMI (r = 0.026, p = 0.769). The correlation between TBS and the variables of interest is analyzed using the Pearson correlation coefficient.

BMD, bone mineral density; BMI, body mass index; TBS, trabecular bone score
